# Data-driven system to predict academic grades and dropout

**DOI:** 10.1371/journal.pone.0171207

**Published:** 2017-02-14

**Authors:** Sergi Rovira, Eloi Puertas, Laura Igual

**Affiliations:** Departament de Matemàtiques i Informàtica, Universitat de Barcelona, Gran Via de les Corts Catalanes 585, 08007 Barcelona (Spain); IUMPA - Universitat Politecnica de Valencia, SPAIN

## Abstract

Nowadays, the role of a tutor is more important than ever to prevent students dropout and improve their academic performance. This work proposes a data-driven system to extract relevant information hidden in the student academic data and, thus, help tutors to offer their pupils a more proactive personal guidance. In particular, our system, based on machine learning techniques, makes predictions of dropout intention and courses grades of students, as well as personalized course recommendations. Moreover, we present different visualizations which help in the interpretation of the results. In the experimental validation, we show that the system obtains promising results with data from the degree studies in Law, Computer Science and Mathematics of the Universitat de Barcelona.

## 1 Introduction

Since Bologna Process [1] was introduced, most European Universities have developed a tutoring system to provide their students with mentorship. The responsibilities of the tutor may differ between institutions but his/her main role is to offer personal guidance and advice to the students. Several recent works point out that personalized mentorship is crucial to prevent dropout, and improve their academic performance (see Section 1.1). Actually, decreasing dropout is one of the main current goals in European Higher Education Area (EHEA). The European dropout rate is 30% according to the publication Education at a Glance (EAG) [2]. In Spain, the dropout rate stands between 25% and 29% according to [3] and in the University of Barcelona, the dropout rate from 2009 to 2014 was around 20% accordingly to [4].

Moreover, universities now offer a broader range of specialized degrees than ever before (minors, double degrees, interdisciplinary and inter-university masters). Therefore the number and variety of students has increased and consequently has made tutorship a more challenging task. All data recorded for every student, such as grades, hours of study and previous academic achievements can be useful information for the tutor but it is not always available. Even if this data is gathered and the tutors have access to it, the sheer size of information is unmanageable by them.

In this context, an automatic tool to process and analyze the accumulated annual curricular data of the students could be extremely helpful for the tutor task [5, 6]. In this work, we present a data-driven system, based on machine learning techniques, for two different tasks: 1) the early prediction of student dropout and 2) the prediction of subsequent course grades for every student, as well as personalized course recommendations. The early dropout prediction indicates those students who are in most need of help. Tutors can focus on them and thus, increase their motivation and performance. Moreover, the course final grade predictions and course recommendations are all information useful to provide personalized enrollment guidance and orientation. Tutor can provide information on the courses that a particular student may enroll on each academic year which will most likely result in success.

For the first task, we compare five state-of-the-art classifier methods: Logistic Regression [7], Gaussian Naive Bayes [8], Support Vector Machines [9], Random Forest [10] and Adaptive Boosting [11], with the aim of providing as much inside to the techniques as possible. For the second task, we compare three methods: Collaborative Filtering Recommendation System [12], Linear Regression [13] and Support Vector Regression [14]. For all these methods we consider the student grades as feature vector, since they are significant and easy to objectively measure and update every year. We extensively validate the proposed methods and select the approach with the best performance. We obtain promising results for the degree studies in Law, Computer Science and Mathematics at the University of Barcelona. We also present new visualizations for the interpretation of the different results, which includes student trends in behavior and academic preferences, providing a rich seam of information to tutors and heads of departments in universities. Also, this would enable them to take immediate action to improve their students’ welfare and academic performance which in turn, would prevent students dropping out.

Summarizing, the presented system, techniques and visualizations constitute a tutor tool for evaluating dropout intention and predicting grades which can be easily adapted to any degree study and updated annually. This tool unveils information about the students, therefore it must be confidential and restricted to tutors and heads of department. This limitation tries to avoid any stigmatization of the students by their professors.

To the best of our knowledge, this is the first work which applies several machine learning techniques to predict academic grades and dropout. Previous work has focused on statistical approaches to study the dropout (see Section 1.1). Statistical models are based on assumptions drawn from the underlying problem. If these assumptions are wrong the predictive power of the model will be poor. A statistical analysis is superior to machine learning techniques when trying to understand the variables involving the problem. However, machine learning models are better when it comes to predictive performance because they are not based on assumptions over the problem but over the provided data. Adaptability is another advantage of machine learning techniques over statistics. Taking the dropout problem into consideration, if student performance factors vary over time (difficulty of the courses for example) the assumptions of a statistical model could become obsolete. However, a machine learning model would easily adapt to the new data.

The rest of the document goes as follows: in next subsection, we review relevant previous work in dropout analysis. In Section 2, we present the used data and methods. In Section 3, we experimentally evaluate the proposed methods and discuss the new visualizations. Finally, in Section 4 we present our conclusions and future work.

### 1.1 Related work

Several recent works study the causes related to dropout intention [3, 15–23]. In the majority of these works the dropout rate is defined as the number of students who register for a course and did not formally enroll again for the next two consecutive academic years. This definition is also used in the University of Barcelona and in this paper. Paper [22] states that the study of dropout should take into account two different situations: leave the university system or withdraw from the actual studies but changing to another faculty or institution. More precisely, this distinction does not allow us to better identifying students that have problems with their studies than using the first definition.

The aforesaid studies use data from different sources: public databases such as UNEIX (Portal of the Information System for Universities and Research in Catalonia) [3, 18], data from a particular university [16, 17], or collected data by means of ad-hoc interviews/questionnaires [3, 16–21]. These data contains different information: from student demographic characteristics and educational resources to personal opinions on different academic regards. Several of these studies analyzed data from Catalan universities [3, 18–20].

Collecting personal data, other than academic performance, can be useful for predicting dropout intention, but it may be also costly to gather. In our approach, we train our models using the final grade of each course because this data is already tracked by the universities. Moreover, this information is updated periodically and can be taken into account by our models. However, any other kind of valuable information can be easily added to the models in case of it being provided by the university.

In most of previous studies the variables considered to be predictive of dropout intention are related to the student educational background, his/her actual performance at the university and socioeconomic factors. A wide range of approaches are used to identify and validate the importance of such variables for dropout intention prediction. The author of [15] was the first to focus on the dropout problem and encourage the research on this issue. In [17], a statistical descriptive analysis is used. This study concludes that previous academic performance, first year academic performance, class attendance and enrollment date are variables that are directly linked to dropout. In [19, 20] the authors study dropout intention and learning outcomes simultaneously and create a conceptual model that directly relate the two concepts. The conclusion drawn from this research is that the level of academic satisfaction is important to predict dropout intention. Another approach is adopted in [24] where the authors perform dropout intention prediction using logistic regression with categorical variables such as level of studies of the parents, parents occupation, sex and first year academic performance. Our contribution uses also machine learning techniques, but comparing five different classification models to predict dropout intention.

After analyzing the explanatory indicators of dropout intention, the studies mentioned above suggest different actions that could be performed to reduce dropout rates. For instance, according to the authors of [16, 17], fomenting class attendance and participation, collecting and storing information of the students and developing a program for new students are essential tasks that a university could perform to reduce dropout rates. In [19, 20] it is mentioned that increasing the level of satisfaction with the university experience and the cognitive outcomes would help to reduce dropout rates. To focus on the quality of educational resources and lectures as well as seeking a realistic expectation held by the students before matriculation are among the main tasks to reduce dropout rates. The study in [18] states that there exists moments of special relevance when facing the decision to drop out and that there is a need to provide personal and academic guidance to the students. In [3], the study concludes that an improvement of vocational counseling practices along with mentorship programs would benefit universities and reduce dropout rates. Similar suggestions are stated in other studies such us [21]. The tool presented in this paper could assist universities to implement these suggestions more easily.

Our study is also related to previous works on the educational field as the one presented in [25], where several prediction techniques in data mining are implemented to assist educational institutions with predicting students’ grade averages at graduation time; and the study in [26], which identifies some of the factors that influence the probability of successfully pass a first course in Mathematics by using a classic logistic regression model (logit) and, an asymmetric Bayesian logit model. Two other related works are those presented in [5] and [6]. The former offers a data-driven system to personalize the communication between students and instructors for large STEM (*Science, Thechnology, Engeneering and Mathematics*) introductory courses. The latter studies the difference in motivation and academic self-concept between first-year college students of STEM courses depending on their gender.

## 2 Materials and methods

### 2.1 Data gathering and cleaning

To conduct our research, we have gathered data from a total of 4,434 students who studied the degree in Law (3,463), Mathematics (516) or Computer Science (455) in the University of Barcelona (UB) between the years 2009 and 2014. See S1 File in Supporting Information for the Mathematics and Computer Science degree data and S2 File in Supporting Information for the Law degree data.

Both the degree in Computer Science and the degree in Mathematics consist of 4 academic years with 10 courses each. The degree in Law consists of 4 academic years, the first one has 10 courses and the remaining 3 years have 8 courses each. The collected information consists of the final grade of the different courses taken by the students during their academic years. All data has been gathered by internal personnel of the UB and it is now available under request (see data availability statement). The values in the data set fall in the range between 0 and 10, although we have also missing data, indicated with *NaNs* (Not a Number). Our interpretation for a missing value (NaN) is that a student has not studied that particular courses yet. Whereas our interpretation for zeros is that a student has enrolled to that particular courses but has not completed the necessary tasks or exams to acquire a final grade.

#### Data cleaning

We clean the data according to the following three criteria:

- Students with 5 or more missing values in an academic year are removed from the original data set.
- Students with a mean grade inferior to 2 points out of 10 in an academic year are removed from the original data set.
- Students who do not follow the standard enrollment procedure are removed from the data set. For instance, a student who enrolls to more than 10 courses in an academic year falls in this category.

All the data that have been removed from the original data set corresponds to rare cases that would bias the results of the models.

### 2.2 Dropout prediction

We try to answer the following question: is it possible to predict if a student will enroll to University in the second or third year given information of the first academic year?

As stated in [18], 58% of the dropouts occur in the first year of university studies. Thus we consider suitable to constraint our research to study first-year dropouts.

Table 1 shows the percentage of students who dropped out after their first and second academic year in the UB data set for Law, Computer Science and Mathematics. As expected, the percentage in the first year is the highest.

**Table 1:** Dropout.

	First year	Second year	Total
*Law*	11.6%	6.6%	18.2%
*ComputerScience*	22.9%	10.4%	33.3%
*Mathematics*	33.1%	15.7%	48.8%

The dropout problem is an imbalanced binary classification problem which can be tackled by the following two-step procedure:

#### Step 1: Feature vector definition and data pre-processing

Each student in the data set is described using an n-dimensional vector consisting of the grades of each course of a given academic year. For Computer Science and Mathematics *n* = 10 for all the academic years and for Law *n* = 10 for the first academic year and *n* = 8 for the rest. We discard to include other information in the feature vector, such as the admission score, to make the system less dependent to external factors (as explained in Section 1.1). In order to properly train the classifiers we have used Synthetic Minority Over-sampling Technique (SMOTE) [27] to balanced the dataset.

#### Step 2: Classification

We train 5 classifiers: *Logistic Regression* (LR) [7], *Gaussian Naive Bayes* (GB) [8], *Support Vector Machine* (SVM) [9], *Random Forest* (RF) [10] and *Adaptive Boosting* (AdaBoost) [11] using the feature vector of the training set samples. We choose these 5 classifiers since they are state of the art techniques which use different approaches to solve a classification problem. We provide a brief explanation of each model in the following paragraphs:

Logistic Regression (LR) is a linear model for classification. The probabilities describing the possible outcomes of each feature vector are moduled using the logistic function (or sigmoid function) that in its standard form is:

$$f\left(x\right)=\frac{1}{1+e^{-x}}$$

Naive Bayes Classifier (NB) is a conditional probability model based in Bayes’ theorem. Given a n-dimensional feature vector (*x*_1_, …, *x*_*n*_) and a classification class *C*, the algorithm computes *p*(*C*|*x*_1_, …, *x*_*n*_) using the Bayes’ theorem. In practice, independence between features is assumed. Combining this with a decision rule, the classification $\hat{y}$ for a feature vector (*x*_1_, …, *x*_*n*_) is done as follows:

$$\hat{y}=\underset{j\in \{1,2\}}{arg max}p\left(C_{j}\right)\prod_{i=1}^{n}p\left(x_{i}|C_{j}\right).$$

Support Vector Machine (SVM) is a classifier based on the idea of separating data using hyper-planes. More specifically, it consider a set of *n*–dimensional feature vectors as points in the *n*–dimensional real euclidean space. It supposes that each point is associated with one class (0 or 1) and solves the problem of separating the points from each class by finding the hyper-plane which is at the largest distance from both points of class 0 and points of class 1.

Random Forests Classifiers (RF) are an ensemble learning technique that works by constructing a multitude of Decision Trees [28] and outputs the mode of the classes of the individual trees. This model is trained using *Feature Bagging*.

Adaptive Boosting (AdaBoost) is a boosting technique which combines multiple weak classifiers *h* into a strong classifier *H*. A weak classifier is a model that performs slightly better than random guessing. The combination of *T* weak classifiers is performed as follows:

$$H\left(x\right)=sign\;\left(\sum_{t=1}^{T}\alpha_{t}h_{t}\left(x\right)\right),$$

where $\alpha_{t}=\frac{1}{2}\ln(\frac{1-\epsilon_{t}}{\epsilon_{t}})$ and *ϵ*_*t*_ is the exponential loss function value for the weak learner *h*_*t*_. We choose as weak classifier a *Decision Stump* [29].

### 2.3 Final grade prediction

As mentioned in the introduction, the tutor can benefit from knowing which courses from the next academic year will be more difficult for a concrete student in order to offer useful enrollment guidance. With the final goal of providing the tutor with an objective criteria to evaluate course difficulty, we compute an approximation of future grades of the students, i.e. grades of the subsequent academic year.

More precisely, we consider the problem of predicting future grades for a particular student given previous ones. To solve this problem we implement a Collaborative Filtering Recommendation system with baseline adjustment [12] due to its scalability and flexibility to handle missing values. This approach has been compared with regression models such us Linear Regression [13] and Support Vector Regression [14], giving those less accurate results than the presented by the Recommender system (see section 3.4.2). The feature vector used is the same as in section 2.2.

In general, a recommendation system works by finding similarities between the rows of a sparse matrix and predicting the missing values using data from the same matrix. In our particular case, the rows of the matrix correspond to the grades of students for each courses of a degree. The recommender systems works under the assumption that if two courses are similar and a student does not have a grade for one of the courses then it is valid to predict this unknown value using his/her grade of the similar one. An off-the-shelf content-based recommendation system for our data set would predict an unknown grade for a particular student *s*_*i*_ and a particular course *c*_*i*_ by computing a weighted average of the student’s grades for similar courses to *c*_*i*_. This naive approach would not take into account important inherent characteristics of our data set such as global mean grade, student grade deviation and course grade deviation. The recommendation approach proposed in [30] adapts perfectly to the nature of our problem. In technical terms, the model works as follows: We consider a matrix R of size *c* × *s*, where *c* corresponds to the number of courses and *s* to the number of students. Then, the next baselines are computed:

$$b_{ui}=\mu +b_{u}+b_{i}$$

$$b_{i}=\frac{\sum_{u\in R_{i}}\left(r_{ui}-\mu \right)}{|R_{i}|}$$

$$b_{u}=\frac{\sum_{i\in R_{u}}\left(r_{ui}-\mu -b_{i}\right)}{|R_{u}|}$$

where, the set *R*_*i*_ consists of the students who have studied course *i*. Similarly, the set *R*_*u*_ consists of the set of courses studied by the student *u*. *μ* is the mean of the matrix R and *r*_*ui*_ is the value in position (*u*, *i*) of the matrix *R*.

To make a prediction for a student *u* and course *i* we use Eq (1) as in [30]:

$$r_{ui}=b_{ui}+\frac{\sum_{j\in S^{k}\left(i,u\right)}s_{ij}\left(r_{uj}-b_{uj}\right)}{\sum_{j\in S^{k}\left(i,u\right)}\left|s_{ij}\right|}$$ (1)

where the set *S*^*k*^(*i*, *u*) consists of the *k* courses studied by the student *u* that are most similar to the course *i*. This set of similar courses is computed by the KNN algorithm with Pearson similarity as distance measure. The similarity between a course *i* and a course *j* is denoted by *s*_*ij*_.

#### 2.3.1 Course ranking

Using the approach presented in the previous Section, we provide an approximation of future grades of students. This is a very rich information for tutors, however we find that a ranking of courses for each student can be even more readable and useful. We want to give the tutor a criteria to identify what courses will be more difficult for a particular student and to do so, we do not need to know the exact grade for each course.

Given a grade *g* we apply standard Spanish thresholds to define four different discrete grades *A* > *B* > *C* > *D*:

$$f\left(g\right)≔\left\{\begin{array}{cc} D, & \text{if}\;g<5\\ C, & \text{if}\;5\leq g<7\\ B, & \text{if}\;7\leq g<9\\ A, & \text{if}\;9\leq g\leq 10 \end{array}\right.$$ (2)

We use these quantized grades to perform the ranking. Finally, we sort all courses of a student in descending order. With the new arrangement of the predicted grades, the tutor acquires extra information about a student at a glance. This can help the tutor to guide students using personalized information about them.

## 3 Experiments

In this Section we explain the performed experiments for evaluating which are the best models to predict dropout among the 5 described in Section 2.2. We also explain the experiments performed to assess the performance of our recommender system predicting grades and compare it with two standard methods described in Section 2.3. Moreover, we provide useful data visualization for a better understanding and interpretation of the results.

### 3.1 Implementation

All models and methods used in the experiments are implemented in Python. In particular, Pandas python library [31] is used to manipulate the data and scikit-learn python library [32] is used to implement the machine learning techniques. For data pre-processing we have used the tool presented in [33]. All the code is publicly available in a GitHub repository (https://github.com/pid-ub/pid-UB).

### 3.2 Evaluation metrics

The performance of the classifiers is assessed using the standard measures of accuracy, recall, precision and F1. *Mean Absolute Error* (MAE) score and *Kendall* [34] ranking correlation are used to evaluate the recommender system.

#### 3.2.1 Classifier metrics

The classifier metrics are defined as follows:

$$Accuracy=\frac{tp+tn}{tp+tn+fp+fn},$$

$$Precision=\frac{tp}{tp+fp},$$

$$Recall=\frac{tp}{tp+fn},$$

$$F1=\frac{2tp}{2tp+fp+fn},$$

where *tp* is true positive (dropout), *tn* true negative (not dropout), *fp* false positive and *fn* false negative. We consider dropout as the positive class and non-dropout as the negative class. Because we want to minimize false negatives (students who drop out are predicted as students who do not drop out) we will select models with the high recall over those with better precision. We will analyze the trade-off between these metrics using F1.

#### 3.2.2 Mean absolute error

To compute the difference between the matrix containing the real grades *R* and the matrix containing the predictions *P*, we use *Mean Absolute Error*:

$$MAE=\frac{1}{\left(c\times s\right)-R_{NaN}}\sum_{i=1}^{c}\underset{R_{i,j}\neq NaN}{\sum_{j=1}^{s}}\left|R_{i,j}-P_{i,j}\right|$$

where *c* is the number of courses, *s* the number of students and *R*_*NaN*_ is the number of missing values in matrix *R*.

#### 3.2.3 Kendall ranking correlation

To evaluate the correlation between to rankings *r*_1_ and *r*_2_ we use *Kendall* *τ* *b* measure [34]:

$$\tau_{t,s}=\frac{P-Q}{\sqrt{\left(P+Q+T)(P+Q+S\right)}},$$

where *P* is the number of concordant pairs, *Q* is the number of discordant pairs, *T* is the number of ties only in *r*_1_ and *S* is the number of ties only in *r*_2_. If a tie occurs for the same pair in both *r*_1_ and *r*_2_, it is not added to either *T* or *S*.

A value close of 1 indicates strong correlation and -1 indicates strong disagreement between the rankings *r*_1_ and *r*_2_.

### 3.3 Evaluation strategy

To evaluate our models we use two different approaches. For dropout classification the data set is split in 60% train and 40% test, training the models using grid search and cross-validation on the training set and evaluating them on the test set. For the evaluation of the recommender system we do not need training and we can use the full data set to perform 5-fold cross-validation.

All the experiments have been performed using data from first and second academic years of each degree due to lack of complete data in the remaining years. Moreover, these are the students in most need of expert advice about enrollment options. Note that all the algorithms are general and the experiments can be conducted for any academic year given that enough data is provided.

### 3.4 Results

Next, we present the results on dropout prediction and grade prediction. We include the most representative graphics among the ones obtained using data from Law, Mathematics and Computer Science degrees. The graphics not contained in this section are provided as supporting information.

#### 3.4.1 Dropout prediction

**Fig 1:**
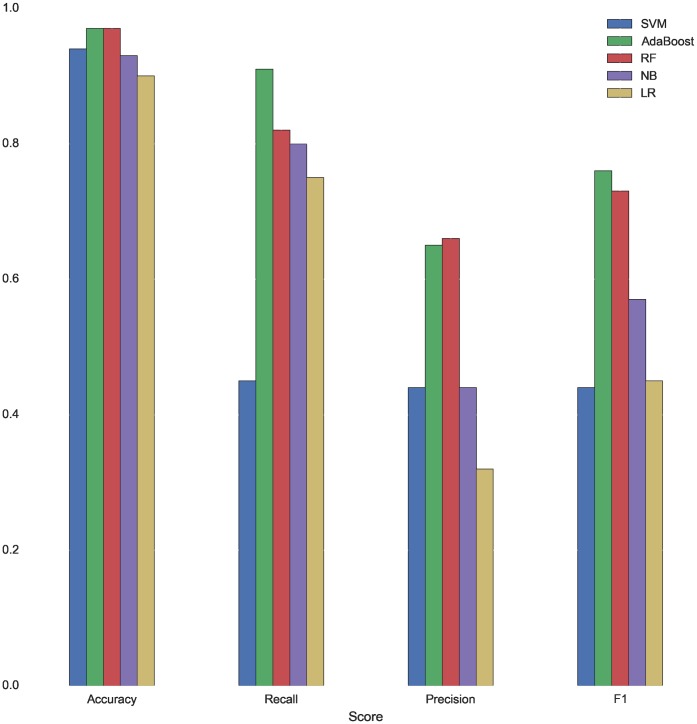
Scores of the models trained for dropout prediction—Law.

Fig 1 summarizes the evaluation of the 5 models trained to predict dropout with data corresponding to the degree in Law. See S1 and S2 Figs in the Supporting Information for the evaluation with data corresponding to the degree in Computer Science and Mathematics, respectively.

**Fig 2:**
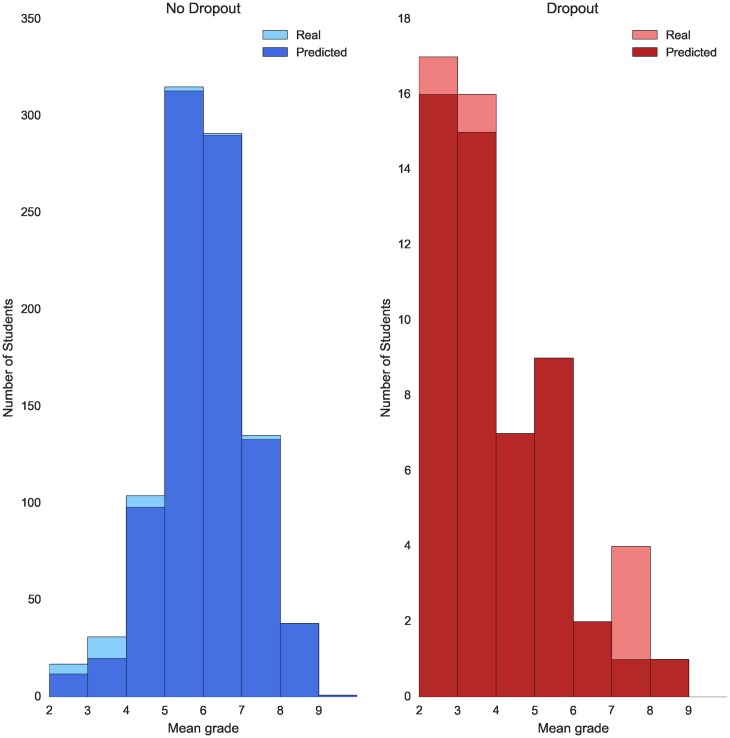
Error dropout prediction by mean grade—Law. Plot showing dropout prediction for both students who do not drop out (blue) and students who drop out (red) grouped by their mean grade after their first academic year. Note that the scale of the two plots is set different for visualization purposes.

Although all the machine learning algorithms reach an accuracy around 90%, RF and AdaBoost perform better when predicting dropout, both obtaining a F1 score of 76% and 73% respectively. AdaBoost reaches a recall score of 91% and Random Forest of 82%. The errors made by AdaBoost are illustrated in Fig 2. In this histogram, we plot the distribution of the students by their mean grade. The figure is divided in two parts. The left plot consists of the predictions made for students who do not drop out after studying their first academic year and the right plot consists of the predictions made for students who drop out after studying their first academic year. The light blue and red colors corresponds to the real distribution of students, whereas the dark colors corresponds to the distribution of students based on the predictions of the classifier. This visualization allows to clearly appreciate the FP errors as the light blue portions of the bars and the FN errors as the light red portions of the bars. It is important to study the predictions made for students with a mean grade between 4 and 6. Without using a classifier, these students would be the most difficult to classify. The model has been able to properly classify all the students with mean grade between 4 and 6 who drop out, making little error identifying those who do not. This implies that the resources provided by the University will be used more efficiently and the students in most need of advise will get access to personalize help more quickly.

**Fig 3:**
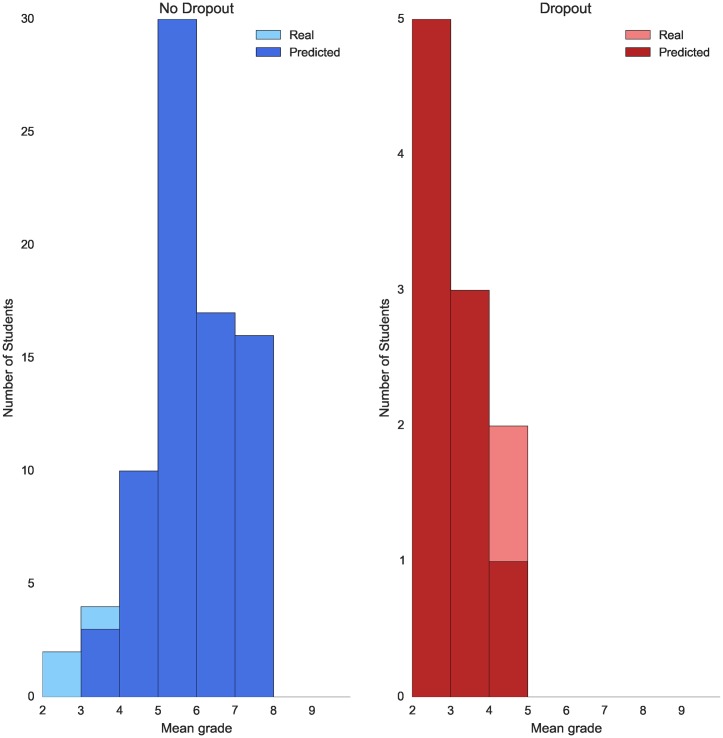
Error dropout prediction by mean grade—Computer science. Plot showing dropout prediction for both students who do not drop out (blue) and students who drop out (red) grouped by their mean grade after their first academic year. Note that the scale of the two plots is set different for visualization purposes.

**Fig 4:**
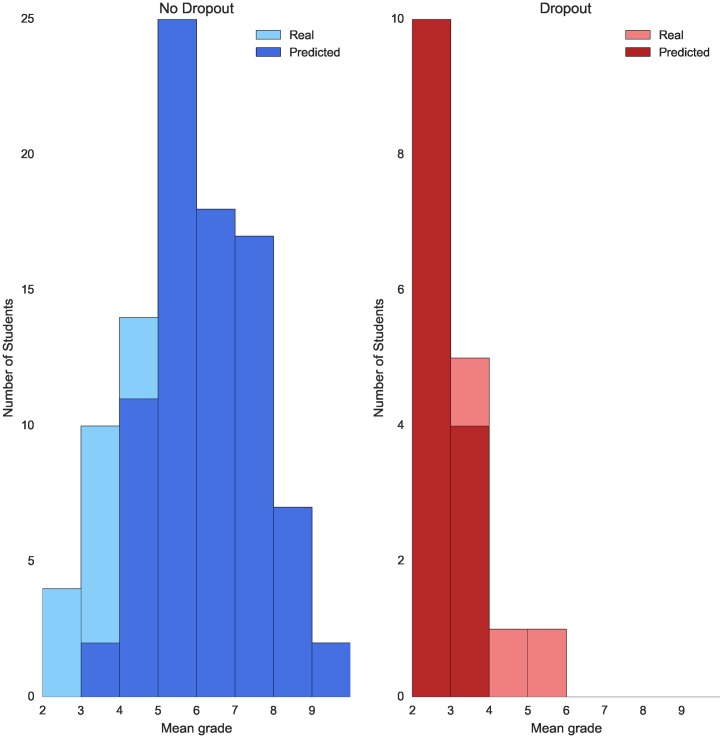
Error dropout prediction by mean grade—Mathematics. Plot showing dropout prediction for both students who do not drop out (blue) and students who drop out (red) grouped by their mean grade after their first academic year. Note that the scale of the two plots is set different for visualization purposes.

For the degree in Computer Science the models that give the best performances are Naive Bayes and Logistic Regression, with a F1 score of 75% and 82% respectively. Regarding the degree in Mathematics, the best models for dropout predictions are also NB and LR with F1 scores of 61% and 60%. The error plots for the degrees of Computer Science and Mathematics are shown in Figs 3 and 4 respectively.

We consider these results to be consequence of the data sets of Mathematics and Computer Science being smaller than the data set of the degree in Law. Given the results of our experiments we think that in order to compute dropout for a small data set (fewer than 1000 samples) it is better to use a non-parametric model such as NB or LR and for larger data sets it is better to train a parametric model like RF or AdaBoost. To sum up, dropout prediction is a problem that requires a rich data set with many samples of academic information. If a degree does not have enough samples a non-parametric model such as NB can be used to correctly classify non-dropout students and the majority of those students in most need of help (mean academic grade between 2 and 4).

Finally, a permutation test over the tested models has been performed and the obtained p-value is 0.0099 for the three data sets, proving that the results are statistically significant.

#### 3.4.2 Grade prediction

After performing 5-fold cross-validation with data from the degree in Mathematics, Law and Computer Science, we obtain the results shown in Table 2.

**Table 2:** MAE score.

Models	Law	Computer Science	Mathematics
*Recommender*	1.215	1.321	1.344
*SV R*	1.313	1.397	1.547
*LinearRegression*	1.219	1.334	1.374

The Recommender is the model giving the best performance. The analysis presented below corresponds to the predictions made by the Recommender.

**Fig 5:**
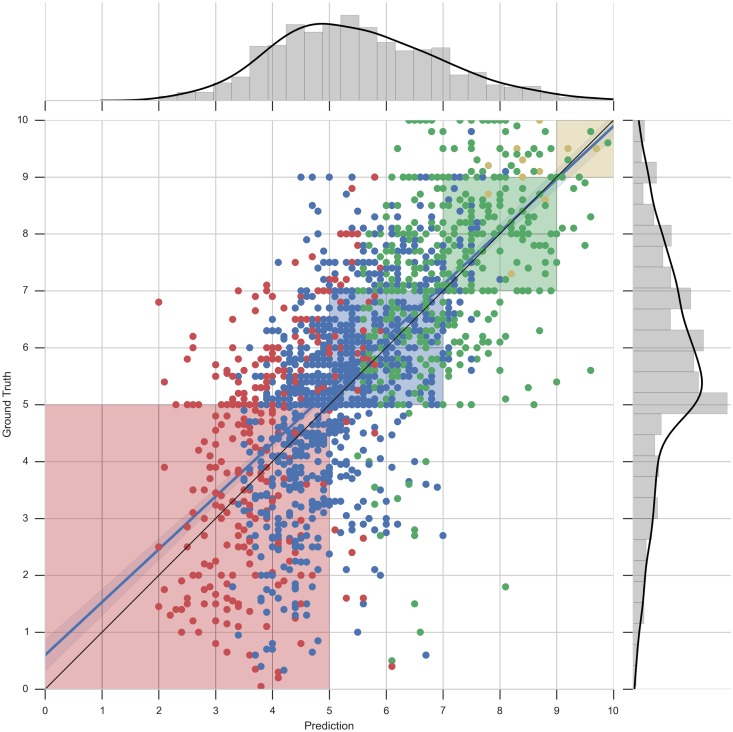
Scatter plot and distribution visualizations of grade predictions—Mathematics. Predicted values against real values for second-year grades for the degree in Mathematics. Each point corresponds to a grade of a student for a particular course. The dots are colored accordingly to the mean grade obtained by the students in the previous academic year. The shaded regions correspond to acceptable errors. The histogram plots show the distributions of the predicted grades (X-axis) and real grades (Y-axis).

It is interesting to notice that for large data sets we obtain a low MAE (1.215 for the degree of Law) and for smaller data sets a higher MAE is obtained (1.321 for Computer Science and 1.344 for Mathematics). In order to visualize how this error is distributed along the predictions, we visualize each predicted grade for second-year courses compared with the real grades of our data set in Figs 5 (Mathematics degree) and 6 (Computer Science degree).

**Fig 6:**
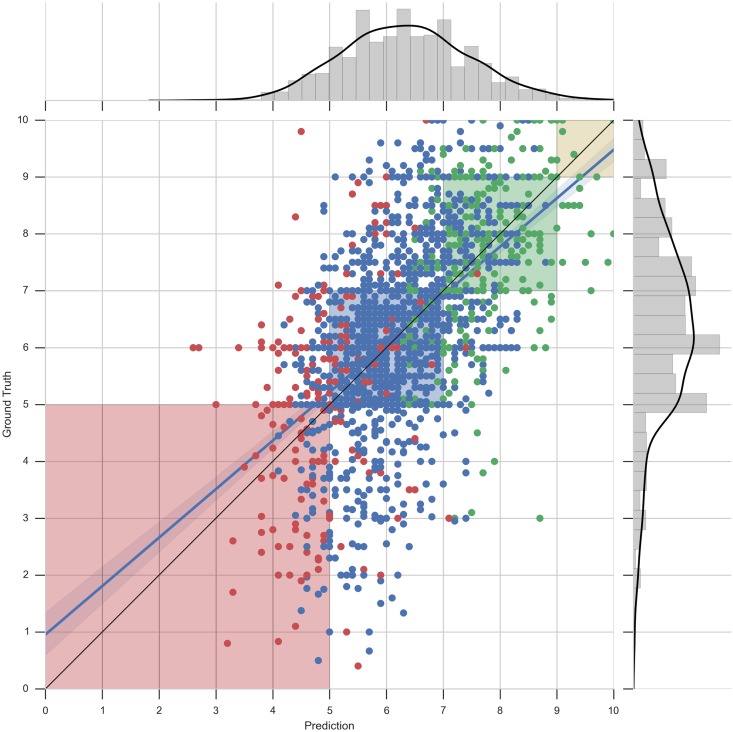
Scatter plot and distribution visualizations of grade predictions—Computer science. Predicted values against real values for second-year grades for the degree in Computer Science. Each point corresponds to a grade of a student for a particular course. The dots are colored accordingly to the mean grade obtained by the students in the previous academic year. The shaded regions correspond to acceptable errors.

These figures are composed of two parts: 1) a central area showing a scatter plot of predicted grades (X-axis) and real grades (Y-axis) of all second year courses, along with a perfect score line (black line) and a best linear regression fitting line (blue line), and 2) two histogram plots showing the distributions of the predicted grades (X-axis) and real grades (Y-axis). The 4 shaded regions of the scatter plot correspond to the areas where the quantized predicted grades (Eq (2)), would be accepted as correct.

Let us comment the graphic in Fig 5. We observe that the linear regression fitting line is near to the perfect score line. The points falling in the white areas of the plot are those wrongly predicted quantized grades. To provide more visual information we have colored each point according to the mean grade obtained by the student in the previous academic year. Each color corresponds to A = yellow, B = green, C = blue, D = red, following thresholds created by Eq (2).

It can be seen that the vast majority of the grades that should fall within the red-shaded region of the plot do so. This means that our recommender system is able to identify the courses that will be the most difficult ones for a student in the next academic year. This is satisfactory, since it is particularly important to rank the most difficult courses properly, where tutor can influence. The dots drawn in the left of each shaded region correspond to courses with a predicted grade lower than the real one while the dots drown in the right hand side of each shaded region correspond to courses with a predicted grade higher than the real one. The number of dots in the first region compared to the number of dots in the second region and the proximity of those to the shaded regions indicates that the model is moderately pessimistic. This characteristic is essential due to the nature of the problem in hand. We prefer to give extra support to a student that would successfully pass a course without help than having the risk of missing a student in need of advice.

We finish the discussion of our predictions by analyzing the distribution of the data set. The histogram plotted along the Y-axis in Fig 5 shows that there are fewer examples of courses which have a grade inferior to 5 than the rest. This explains the tendency of our recommender to give predictions closer to 5.

Fig 6 show the performance of the recommender when predicting grades for smaller data sets. The analysis is done with the data of the degree in Computer Science. Note that the distribution of the data in this plot is more skewed than the previous one (Fig 5), making the predictions of grades even more challenging. It can be seen that the prediction in the red-shaded area is more poorly done than before. The reason for this is that data does not contain many samples of grades between 5 and 0 as shown in the Y-axis histogram. Thus, the recommender does not have enough information to perform a more accurate approximation and it tends to predict following a Gaussian distribution, as shown in the X-axis histogram. Moreover, looking at the colors of the points, one can appreciate that the wrongly predicted grades of the red-shaded area correspond to students who did relatively well in their previous academic year (blue points) and the correctly predicted ones correspond to those students who did badly in their previous year (red points).

See S3 Fig in the Supporting Information for the performance with data corresponding to the degree in Mathematics.

**Fig 7:**
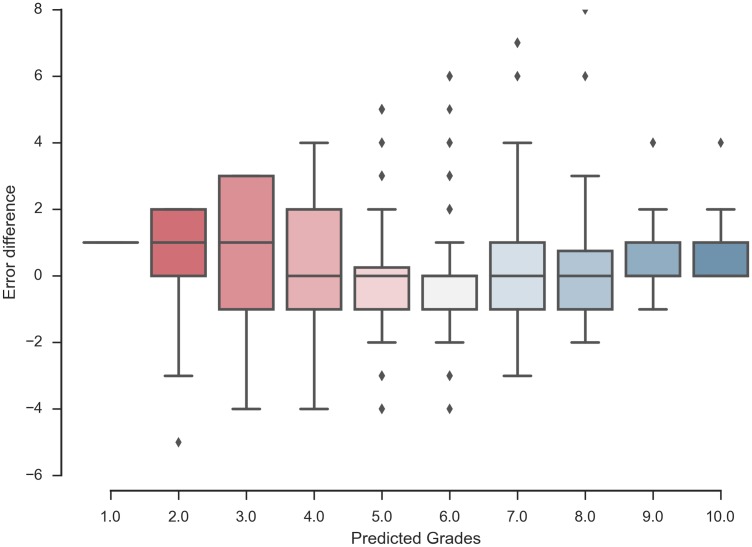
Interpretation graphic for predicted grades errors—Mathematics. Box plot showing the error difference made by the recommender regarding predicted grades of the degree in Mathematics.

We want to provide the tutor with a way to further interpret the data provided by our recommender. Fig 7 shows the spread in the error between the predicted grades and the real ones for the degree in Mathematics. We compute the difference between predicted grades and real grades. A positive value of the difference means that the recommender has predicted a higher grade than the real one and a negative value means the opposite. This figure shows how to interpret a prediction of a given value made by the recommender. For instance, if the recommender has predicted a 10, this value is likely to be any number between 8 and 10 since the deviation is 2. We can observe that the most accurate predictions are those made for grades higher than 5, being 6 the most unreliable prediction of all (presence of outliers).

See S4 and S5 Figs in the Supporting Information for the interpretation of predicted grades errors for the degree in Law and Computer Science, respectively.

Finally, we compute the Kendall measure and provide another way to elucidate the ranking performance.

A Kendall correlation score of 0.29 is obtained for the degree in Computer Science, meaning that there is moderate agreement between the predicted ranking and the real one. A Kendall correlation score of 0.12 and 0.21 for the degrees in Law and Mathematics respectably.

**Fig 8:**
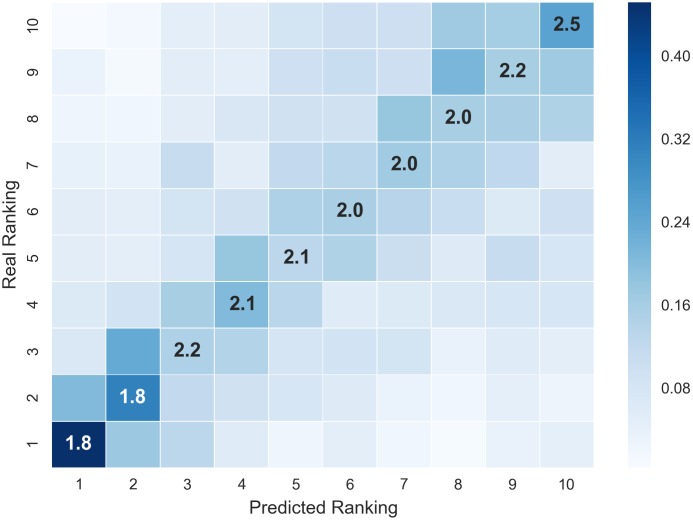
Heat map for ranking evaluation—Computer science. Heat map showing probabilities of ranking correctness for the degree in Computer Science.

The heat map shown in Fig 8 provides a way to interpret the meaning of the obtained Kendall correlation score. The X-axis corresponds to the positions of the predicted ranked courses and the Y-axis corresponds to the real ranked course positions. The intensities of the heat map (in the scale shown in the colorbar of the plot) have the following meaning: The intensities in the diagonal cells illustrates the percentage of the correctly positioned courses. The rest of cells illustrates the error in the ranking. For instance, we can see that in position (1,1) the intensity of the cell corresponds to a value of almost 0.40, which means that in average, 40% of the courses that where predicted to be at the top of the ranking (position 1) were actually at the top in the real ranking (position 1). In other words, given a new ranking, there is a probability of 0.40 that course placed in the first position is predicted correctly. Moreover, the numeric values of the diagonal correspond to the average error for each position in the ranking, i.e, the value 1.8 in position (1,1) means that in average a course ranked in position 1 in the predicted ranking would be likely to be at position 3 in the real one. It is worth to note that the intensities of cells in position 1 and 2 are much higher than the rest. Taking into account that the mean average deviation for positions (1,1) and (2,2) is almost 2 we can deduce that it is likely that the two most difficult courses for a student will be among the top 4 of the predicted ranking. Therefore, the tutor can fairly advise the students to focus on the top 4 ranked courses.

See S6 and S7 Figs in the Supporting Information for the interpretation of predicted grades errors for the degree in Law and Mathematics, respectively.

## 4 Conclusions

In this paper, we have presented a data-driven system to help tutors in the early detection of dropout and the prediction of courses grade as well as courses ranking. We have compared different machine learning methods and selected the ones with the best performance. Our classification system’s results in dropout prediction are promising, obtaining a F1 score of 82%, 76% and 61% for the degrees in Computer Science, Law and Mathematics respectably. Regarding the final grade prediction, our recommender system is able to accurately predict the grade with an mean absolute error of 1.21, 1.32 and 1.34 for the degrees in Law, Computer Science and Mathematics respectively. Moreover, in order to complete the evaluation of the performances we have developed visualization tools to better understand the obtained results. In particular, these visualizations allow to interpret: where the system commits errors on dropout prediction for each degree; how the errors of predicted grades are distributed for each degree; and how correct the ranking is for each degree.

We have only used grades for training our system. This allows to easily adapt the system for other degree studies and universities, while this data is available.

This work prove the power of machine learning techniques in dropout prediction. This complements previous works done in the Educational Sciences community, where Statistical approaches are mainly used for understanding the underlying cause of problems such as dropout intention.

Regarding educational implications, our system can be extremely useful for the tutors, which will be able to know beforehand which students need help and in which subjects. This information will assist tutors in their main task, which is the personalized enrollment guidance and orientation. Moreover, the tutor task will be more guided by means of the presented visualization tools. We expect that this aided tutorial system has an impact in the student motivation, satisfaction and results improvement. In this way, dropout intention could be reduced and student engagement could be increased.

As future work, we will test our system with new students’ data from UB during the next academic years. In parallel, we will increase the number of students, variety of degrees, branches and universities involved in the work to validate the system in other scenarios. This would result in a much more complete study. In addition, we would like to analyze student profiles by means of different clustering techniques to better identify general characteristics of the students. Finally, we intend to build a practical system based on the developed tools to be part of the teaching platform in UB.

## Supporting information

S1 Fig Scores of the models trained for dropout prediction—Computer science. (TIF)

S2 Fig Scores of the models trained for dropout prediction—Mathematics. (TIF)

S3 Fig Scatter plot and distribution visualizations of grade predictions—Law. Predicted values against real values for second-year grades for the Degree in Law. Each point corresponds to a grade of a student for a particular course. The dots are colored accordingly to the mean grade obtained by the students in the previous academic year. The shaded regions correspond to acceptable errors. The histogram plots show the distributions of the predicted grades (X-axis) and real grades (Y-axis).
(TIF)

S4 Fig Interpretation graphic for predicted grades errors—Law. Box plot showing the error difference made by the recommender regarding predicted grades of the degree in Law.
(TIF)

S5 Fig Interpretation graphic for predicted grades errors—Computer science. Box plot showing the error difference made by the recommender regarding predicted grades of the degree in Computer Science.
(TIF)

S6 Fig Heat map for ranking evaluation—Law. Heat map showing probabilities of ranking correctness for the degree in Law.
(TIF)

S7 Fig Heat map for ranking evaluation—Mathematics. Heat map showing probabilities of ranking correctness for the degree in Mathematics.
(TIF)

S1 File Data from mathematics and computer science degrees. Grades of students from Mathematics and Computer Science degrees (2009–2014) in CSV format file.
(CSV)

S2 File Data from law degree. Grades of students from Law degrees (2009–2014) in CSV format file.
(CSV)
