# Supplementary figures and images for: Data-driven system to predict academic grades and dropout
Source: PLoS One. 2017 Feb 14;12(2):e0171207. doi: 10.1371/journal.pone.0171207 (PMC5308611; doi:10.1371/journal.pone.0171207)

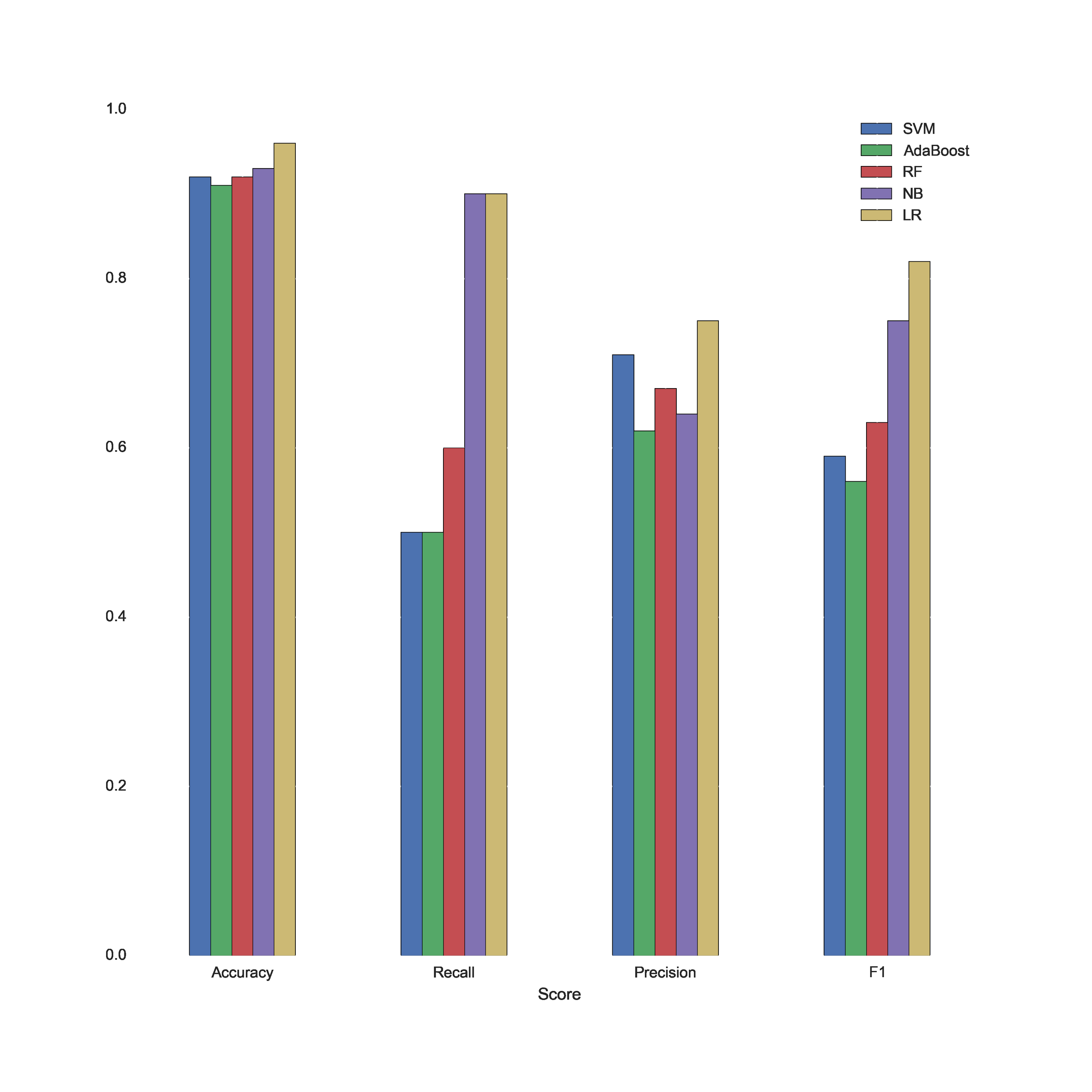

Supplement: S1 Fig — (TIF) [file pone.0171207.s001.tif]

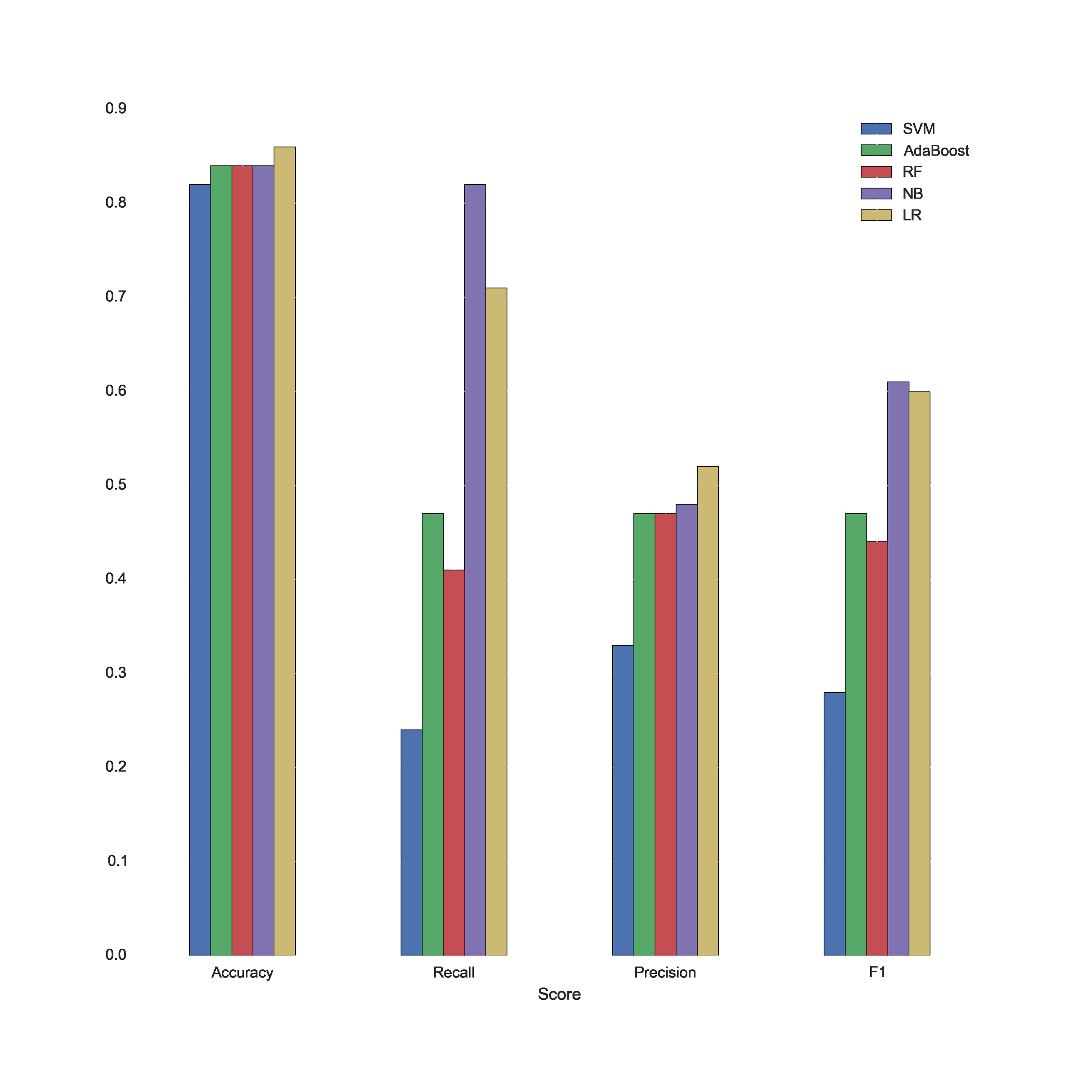

Supplement: S2 Fig — (TIF) [file pone.0171207.s002.tif]

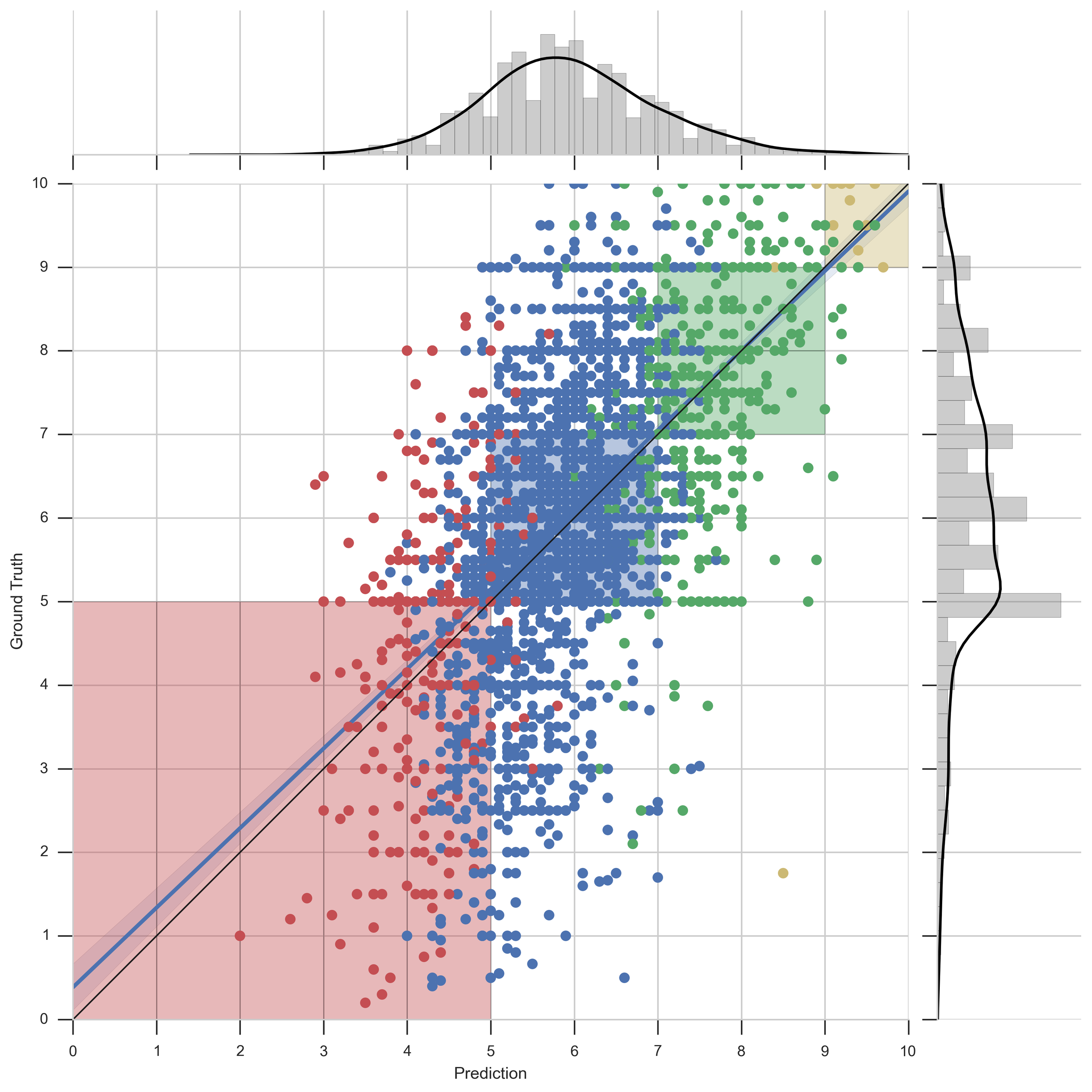

Supplement: S3 Fig — Predicted values against real values for second-year grades for the Degree in Law. Each point corresponds to a grade of a student for a particular course. The dots are colored accordingly to the mean grade obtained by the students in the previous academic year. The shaded regions correspond to acceptable errors. The histogram plots show the distributions of the predicted grades (X-axis) and real grades (Y-axis). (TIF) [file pone.0171207.s003.tif]

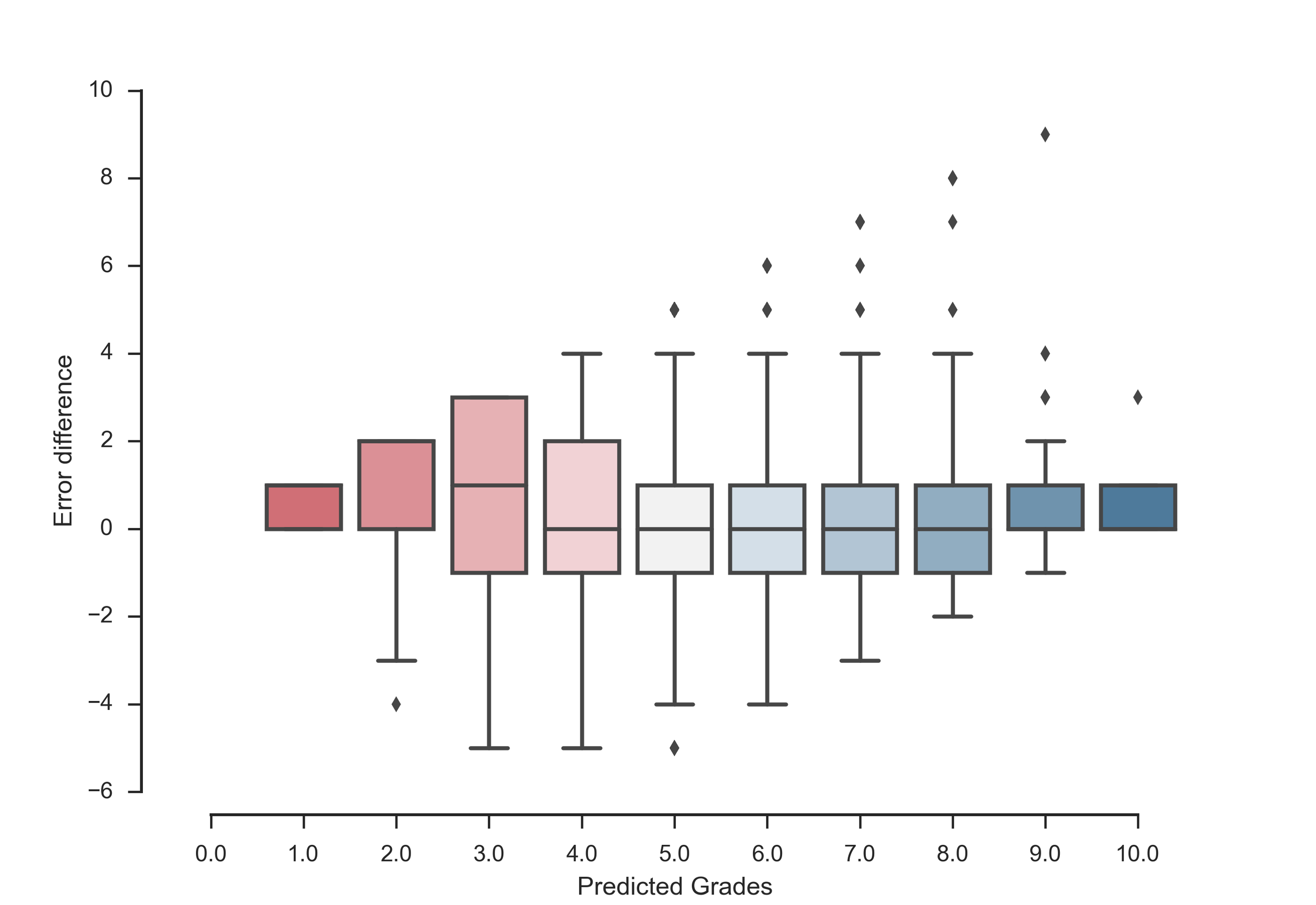

Supplement: S4 Fig — Box plot showing the error difference made by the recommender regarding predicted grades of the degree in Law. (TIF) [file pone.0171207.s004.tif]

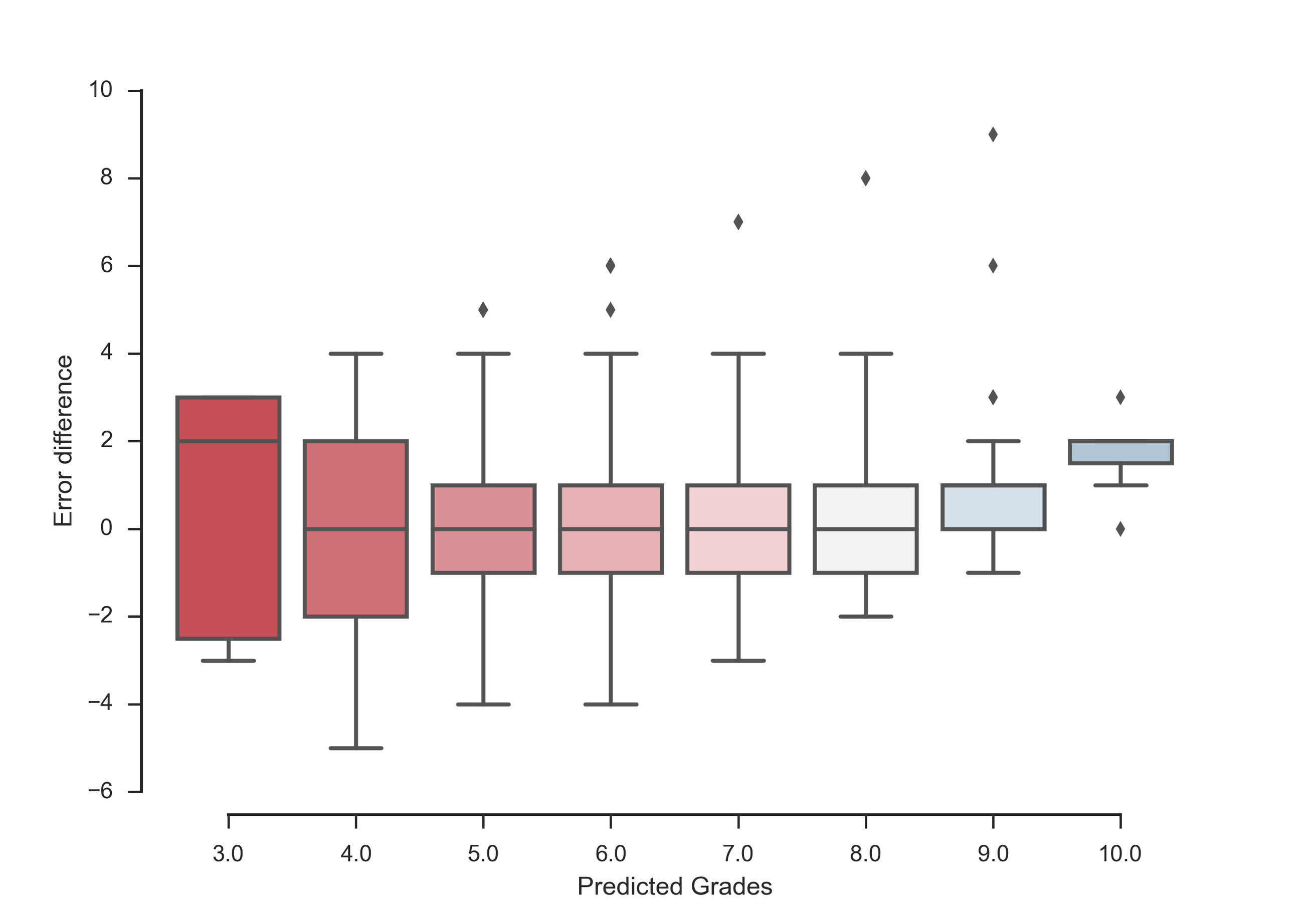

Supplement: S5 Fig — Box plot showing the error difference made by the recommender regarding predicted grades of the degree in Computer Science. (TIF) [file pone.0171207.s005.tif]

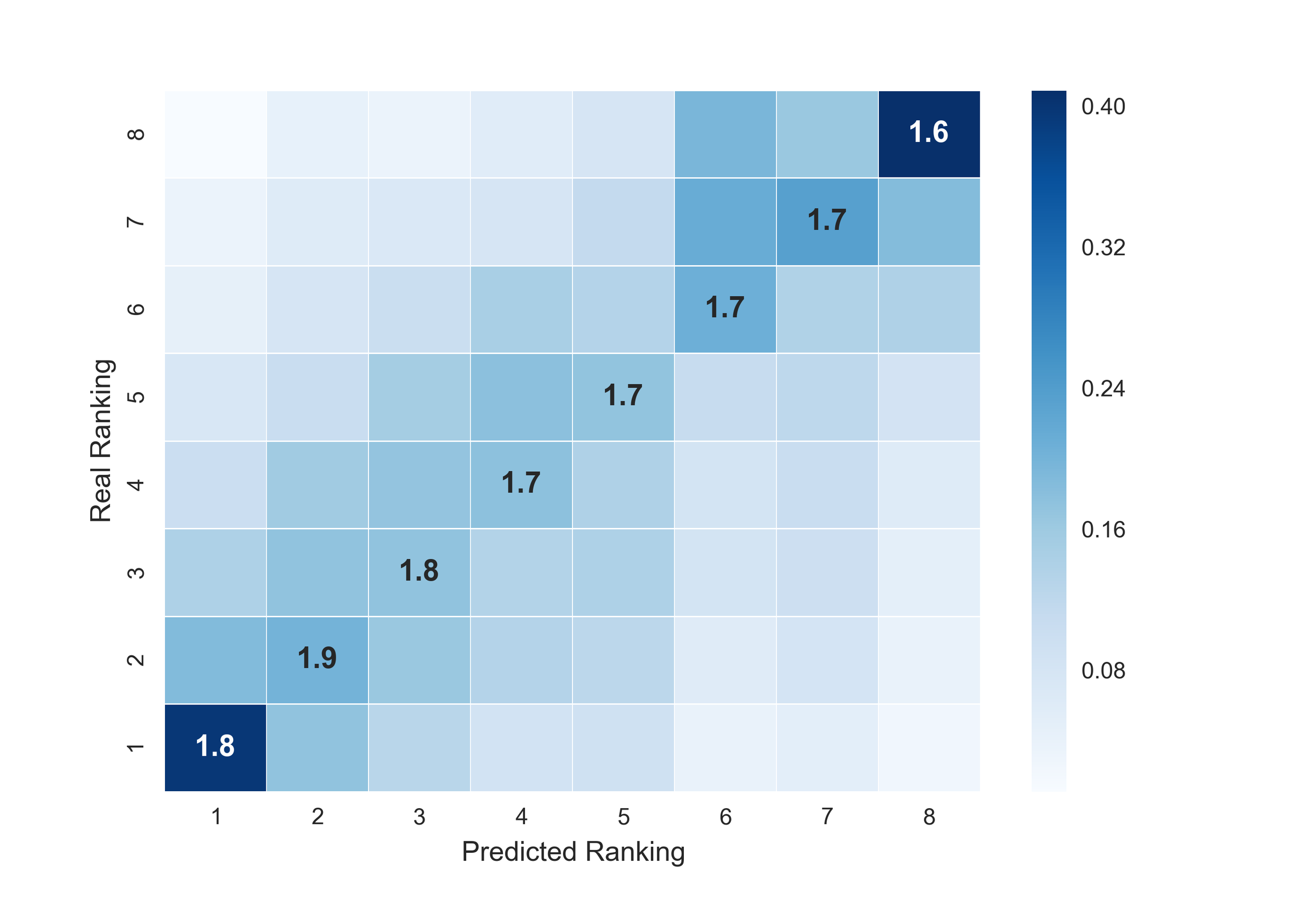

Supplement: S6 Fig — Heat map showing probabilities of ranking correctness for the degree in Law. (TIF) [file pone.0171207.s006.tif]

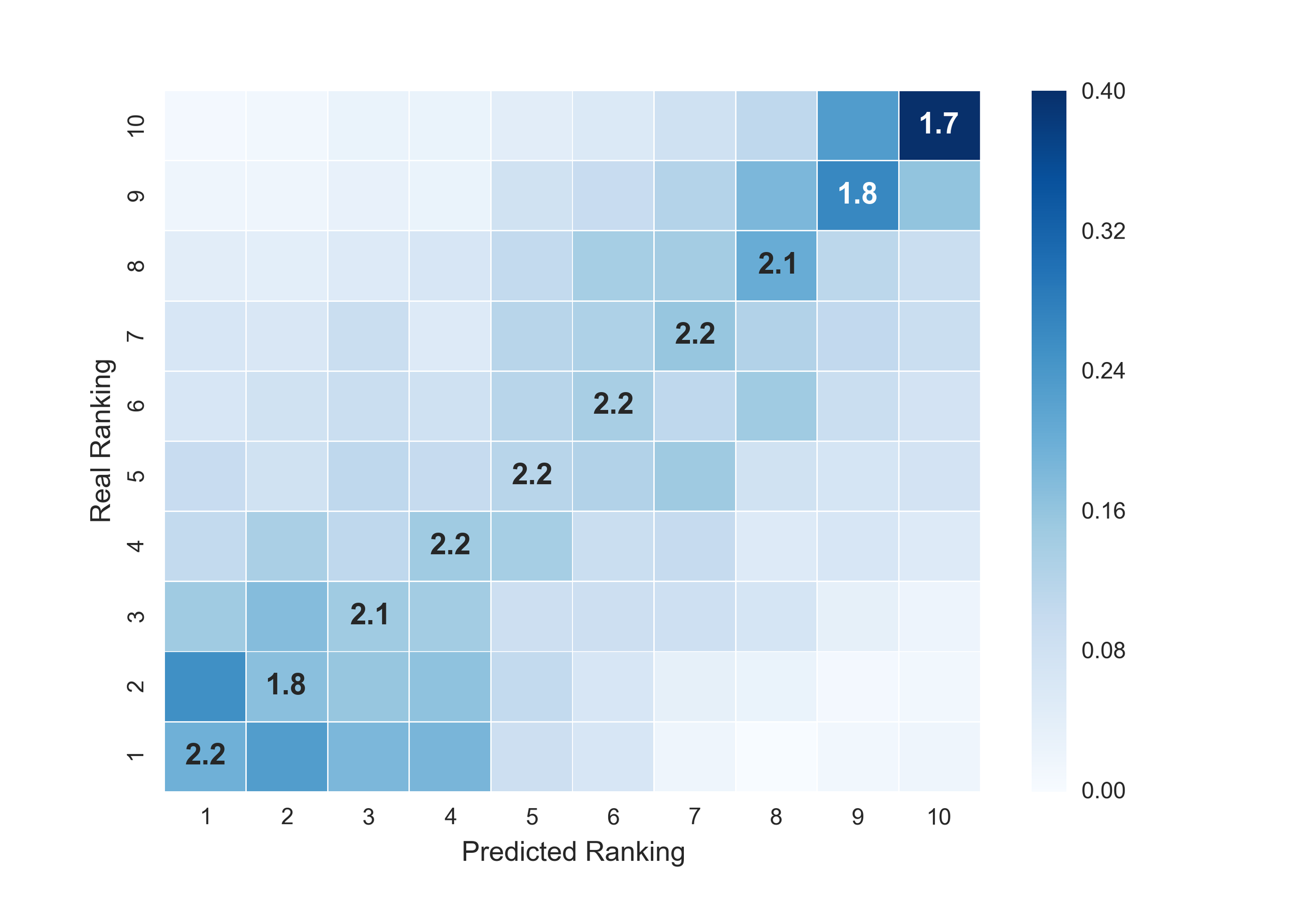

Supplement: S7 Fig — Heat map showing probabilities of ranking correctness for the degree in Mathematics. (TIF) [file pone.0171207.s007.tif]
